# Enhanced Cadmium Accumulation and Tolerance in Transgenic Hairy Roots of *Solanum nigrum* L. Expressing Iron-Regulated Transporter Gene *IRT1*

**DOI:** 10.3390/life10120324

**Published:** 2020-12-03

**Authors:** Peng Ye, Menghua Wang, Teng Zhang, Xiaoyu Liu, He Jiang, Yaping Sun, Xiyu Cheng, Qiong Yan

**Affiliations:** 1 College of Life Sciences and Bioengineering, School of Science, Beijing Jiaotong University, Beijing 100044, China; 17121611@bjtu.edu.cn (P.Y.); 18121618@bjtu.edu.cn (M.W.); 3120160574@bit.edu.cn (T.Z.); 20121609@bjtu.edu.cn (X.L.); 17121607@bjtu.edu.cn (Y.S.); 2 Shangrao Municipal Ecological Environment Bureau, Shangrao 334000, China; river99633@126.com

**Keywords:** cadmium accumulation, transgenic *Solanum nigrum* L., hairy roots, iron-regulated transporter gene, cadmium stress response

## Abstract

*Solanum nigrum* L., a hyperaccumulator of cadmium (Cd), is regarded as a promising candidate for phytoremediation of heavy metal pollution. In the present study, the hairy roots of *Solanum nigrum* L. were selected as a model plant system to study the potential application of Iron-regulated Transporter Gene (*IRT1*) for the efficient phytoremediation of Cd pollution. The transgenic hairy roots of *Solanum nigrum* L. expressing the *IRT1* gene from *Arabidopsis thaliana* were successfully obtained via the *Agrobacterium tumegaciens*-mediated method. Expression of *IRT1* reduced Cd stress-induced phytotoxic effects. Significantly superior root growth, increased antioxidant enzyme activities, decreased reactive oxygen species (ROS) levels, and less cell apoptosis were observed in the transgenic hairy roots of *Solanum nigrum* L. compared to the wild-type lines under Cd stress. Enhanced Cd accumulation was also carried out in the transgenic hairy roots compared to the control (886.8 μg/g vs. 745.0 μg/g). These results provide an important understanding of the Cd tolerance mechanism of transgenic *IRT1* hairy roots of *Solanum nigrum* L., and are of particular importance to the development of a transgenic candidate for efficient phytoremediation process.

## 1. Introduction

Resource, energy, public health, food safety, and environmental problems are of great importance for sustainable development [1,2,3,4,5,6,7,8,9]. Cadmium (Cd) is one of the most toxic heavy metals that produces a high health risk to humans. Serious Cd pollution in soils mainly results from excessive application of fertilizer and sewage sludge in agricultural soils, industrial activities, and so on [1,2]. High Cd intake by the food chain may produce serious fibrosis, tissue inflammation, and even cancers in humans [10,11,12,13]. Therefore, it is urgent to develop an efficient strategy to eliminate Cd from soils. To overcome the heavy metal pollution problems in soils, various techniques have been developed such as chemical immobilization, chemical stabilization, land filling using fresh soil, soil washing, etc. [1,2]. The main drawbacks of these methods are the high cost, destruction to the soil, and unpromising efficiency [1,2]. Phytoremediation technology is increasingly considered as a cost-effective candidate for reducing Cd pollution [8,9,10].

Heavy metal pollutants cannot be degraded by most of these candidate plants, hence making the removal through absorption, transportation, and accumulation in plant biomass during phytoremediation processes [8,9,14,15,16,17]. A number of hyperaccumulators of Cd (e.g., *Brassica campestris* L., *Solanum nigrum* L.), which show relatively high Cd tolerance and accumulation levels compared to common plants, have been identified in previous studies [15]. However, the industrial application of this emerging technology using natural metal hyperaccumulator plants is still limited due to their obvious disadvantages including accumulating efficiency, moderate Cd tolerance, low growth rates [1,2,8,9], etc. Genetic engineering appears to be a promising strategy to improve the phytoremediation efficiency of these plants by overexpressing genes related to metal tolerance, uptake, transportation, and/or detoxification processes to enhance their metal tolerance and accumulation [6,15,16,17]. For example, transgenic tobacco plants overexpressing the rice metal tolerance protein gene OsMTP1, which indicated a superior growth pattern and increased vacuolar thiol content, may obtain increased Cd tolerance by the OsMTP1-mediated enhancement of vacuolar sequestration of Cd [15]. 

The transporter proteins play a vital role in maintaining metal homeostasis and transporting different metals in cells. The iron-regulated transporter (*IRT1*) gene is one of members of the ZIP (ZRT-IRT-like proteins) family, which functions in metal transport (e.g., iron, zinc, manganese, and/or Cd) in different eukaryotic organisms [14,18]. Previous studies show that IRT1 mediates the transport of iron, zinc, and/or manganese in bacteria, yeast, and plants [14]. Zhang reported that *AtIRT1* had a significant up-regulation in the transcription level under Cd stress [6]. Plants overexpressing *IRT1* accumulated higher levels of Cd and Zn than wild-type *Arabidopsis thaliana* [14]. High levels of IRT1 proteins in these transgenic *IRT1* plants indicated the IRT1-mediated Cd-transport and subsequent accumulation in the plant cells and tissues [6,14]. These results indicate that *IRT1* may be a promising candidate gene for genetic engineering modification of the hyperaccumulators of Cd.

Among these candidates for efficient phytoremediation of Cd pollution, *Solanum nigrum* L. is considered as a promising hyperaccumulator [8,17,19,20]. *Solanum nigrum* L. was a newly reported Cd hyperaccumulator, having possible hyperaccumulative mechanisms such as antioxidative defense, praline/phytochelatin, and nitrogen metabolism in leaves [17,19,20]. A previous study also reported that strong Cd tolerance, high cell membrane permeability with intact cell membrane, weak Cd forms in subcellular distribution, and relatively low bioactivity speciation may be reasons for *Solanum nigrum* L. hyperaccumulating Cd [17]. Given the high Cd accumulating ability of natural *Solanum nigrum* L., genetic engineering modification may make it more attractive for future application. However, studies on the development of transgenic *Solanum nigrum* L. expressing promising candidate genes such as *IRT1* for improving Cd-accumulating ability are still limited, and the corresponding molecular mechanism on the absorption, accumulation, and tolerance of Cd in these transgenic plants remains unclear. 

Hairy roots have been proven as an ideal experimental tool for studying the interactions between plant cells and metal ions, and further genetic engineering modification via the *Agrobacterium rhizogenes*-mediated method is quite straightforward for the introduction of improved Cd accumulation traits [10,21,22,23]. Given these attractive advantages, the hairy roots of *Solanum nigrum* L. have been chosen as a model plant system to study the Cd tolerance of hyperaccumulating plants in this work. The main objective of this work was the development of the transgenic hairy roots of *Solanum nigrum* L. expressing *IRT1* for enhancing Cd accumulation. Thereafter, root growth, Cd accumulation, the levels of intracellular ROS, cell damage, and antioxidative response in the transgenic hairy roots were studied. The mechanism behind enhanced Cd tolerance and accumulation in the transgenic *Solanum nigrum* L. was further discussed, hence providing useful information for developing an efficient phytoremediation process with transgenic hyperaccumulators of Cd.

## 2. Materials and Methods

### 2.1. Materials and Media

*Agrobacterium rhizogenes* ATCC15834 was bought from Guangdong province microbial culture collection center. *Solanum nigrum* L. were obtained from Beijing Jiaotong University. The hairy roots were maintained in a *Murashige* and *Skoog* medium (MS) [10]. Pyridoxine flasks containing 50 mL MS medium (with vitamins and sugar) were used for the hairy root culture. These flasks were then incubated at 100 r/min and 28 °C for selected days. The composition of MS medium was as follows: NH_4_NO_3_ 1650 mg/L, MgSO_4_ 180.7 mg/L, KNO_3_ 1900 mg/L, CaCl_2_ 332.2 mg/L, KH_2_PO_4_ 170 mg/L, CoCl_2_·6H_2_O 0.025 mg/L, MnSO_4_·H_2_O 16.9 mg/L, H_3_BO_3_ 6.2 mg/L, CuSO_4_·5H_2_O 0.025 mg/L, FeNaEDTA 36.7 mg/L, KI 0.83 mg/L, ZnSO_4_·7H_2_O 8.6 mg/L, Na_2_MoO_4_·2H_2_O 0.25 mg/L, glycine 2 mg/L, nicotinic acid 0.5 mg/L, pyridoxine HCl 0.5 mg/L, moy-inositol 100 mg/L, and thiamine HCl 0.1 mg/L. 

### 2.2. Development of Wild-Type and Transgenic Hairy Roots of Solanum nigrum L. 

The wild-type hairy roots of *Solanum nigrum* L. were induced by infection of the seedlings with *Agrobacterium rhizogenes* ATCC15834 according to previous method [10,24,25]. The development of transgenic hairy roots of *Solanum nigrum* L. was developed using recombinant *Agrobacterium rhizogenes* ATCC15834 as follows. 

Plasmid pRI101-AN was bought from Takara company. The *IRT1* gene from *Arabidopsis thaliana* (Gene ID: 827713, https://www.ncbi.nlm.nih.gov/gene/827713) was completely synthesized by Sangon Biotech Co., Ltd. (Shanghai, China) in vector plasmid pUC57 with ampicillin resistance. Recombinant plasmid pRI101-*IRT1* was designed by using *Bam*HI and *Sal*I as restriction enzyme sites based on plasmid pRI101-AN (Figure S1). The recombinant plasmid pRI101-*IRT1* was transferred into *Agrobacterium rhizogenes* ATCC15834 competent cells. The sequencing of the recombinant plasmid pRI101-*IRT1* was performed by Beijing Genomics Institute (Beijing, China).

The above recombinant *Agrobacterium rhizogenes* ATCC15834 with plasmid pRI101-*IRT1* was used for the induction of transgenic *IRT1* hairy roots. Firstly, we sheared the 3 weeks’ aseptic seedlings’ leaves, stems, and petioles into 0.5 cm size explants by aseptic surgery, and then put the explants on MS agar medium with 0.6 mg/L NAA (naphthalene acetic acid) and cultured at 28 °C for 48 h without light. After this, the explants were immersed in activated recombinant *Agrobacterium rhizogenes* ATCC15834 for 20 min. They were then put on sterilized wet filter paper at 28 °C for 48 h in dark conditions. Finally, they were placed on MS agar medium containing 500 mg/L cefotaxime sodium to induce hairy roots at 28 °C in dark conditions.

Four-week-old *Solanum nigrum* L. hairy roots with good growth status were transferred into MS liquid medium containing 500 mg/L cefotaxime sodium. The suspended hairy roots were cultivated at 28 °C on a 100-rpm shaking bed. The culture solution was replaced every four weeks. After 5 generations, the dosage of cefotaxime sodium was gradually reduced to zero. Finally, the transgenic hairy roots *Solanum nigrum* L. of stable growth were transferred into MS liquid medium with no antibiotics, and maintained at the liquid culture of the suspension system. 

### 2.3. Determination of IRT1 Gene Expression

For verifying the existence of *IRT1* gene in hairy roots, we amplified the *IRT1* gene by using polymerase chain reaction (PCR) method. We extracted genomic DNA from the transgenic hairy roots of *Solanum nigrum* L. using Plant genomic DNA extraction kit (Tian gen Biotech Co., Ltd., Beijing, China). PCR amplification was then carried out with the primers (Table S1) using the sterile distilled water as blank control. The PCR cycling conditions can be seen from Table S2. PCR products were electrophoresed on a 1% agarose gel, stained in ethidium bromide, and the DNA was visualized under ultraviolet light. The PCR products were sequenced by Beijing Genomics Institute (Beijing, China).

Thereafter, western blotting was used to check the IRT1 protein expression in the transgenic hairy roots according to standard protocols [10,26]. In brief, the fresh hairy roots were ground in liquid nitrogen. Then the total proteins were extracted using a plant protein extraction kit (Beijing ComWin Biotech Co., Ltd., Beijing, China). Then the protein concentrations were determined using a Pierce™ BCA protein assay kit (Thermo Fisher Scientific, Shanghai, China). After that, the Western blot hybridization with Fe^2+^ transporters antibody (Agrisera, Sweden) was then performed for the identification of the IRT1 protein expression. The experiment of Western Blot was conducted with the loading quantity of 20 µg. 

### 2.4. Evaluation of Wild-Type and Transgenic Hairy Roots under Cd Stress

The 0.2 g of wild-type or transgenic hairy roots of *Solanum nigrum* L. were transferred into 50 mL liquid MS medium; they were then incubated at 100 r/min and 28 °C for 21 days. After that, the hairy roots were sub-cultured into liquid MS medium containing various concentrations (0, 25, 50, 75, 100 µM) of CdCl_2_ for 14 days. The hairy roots were collected for analysis of fresh weight, wet weight, ROS levels, etc. These experiments were performed in triplicate.

### 2.5. Determination of Biomass Weight and Cd Content

For fresh weight (FW) determination, the hairy roots were gently pressed on filter paper to remove excess water and weighed. Subsequently, the hairy roots were dried in an oven at 60 °C until a constant weight and dry weight (DW) was measured. The dried hairy roots were ground and digested with mixed acid (nitric acid, perchloric acid = 4:1) by electric stove. The samples were boiled until they were completely digested, and 1% nitric acid was used to quantify the last volume. The Cd content of hairy roots was determined according to the standard method using the atomic absorption spectrophotometer described in our previous studies [27]. The test conditions were: 228.8 nm wavelength, 7 mA lamp current, 113 nm slit width, 30 s, 110 °C drying, and 500 °C ashing.

### 2.6. Observation of ROS Levels and Cell Viability

The ROS levels in the wild-type and transgenic hairy roots were observed based on Carboxy-H2DCFDA fluorescent probe method [28]. In brief, the fresh hairy roots were collected and thoroughly rinsed with distilled water. They were then immersed in the solution of 50 μM carboxy-H2DCFDA fluorescent probes and incubated on a 100-rpm shaking bed for 20 min. Then they were rinsed with distilled water under same conditions and observed in a fluorescent microscope.

The cell viability of the wild-type and transgenic hairy roots was observed based on fluorescent staining with propidium iodide (PI) [15,29]. In brief, the root cultures were immersed in the PI dye solution and stained for 30 min on a shaker at 100 rpm. Then the roots were collected and rinsed with phosphate buffer for 20 min, following which they were observed in a fluorescent microscope.

### 2.7. Analysis of Antioxidant Enzyme Activities 

Different assay kits (NanJing JianCheng Bioengineering Institute, Nanjing, China) were employed to determine the activities of antioxidant enzymes including catalase (CAT), peroxidase (POD), and superoxide dismutase (SOD). In brief, the above enzymes were extracted from the fresh hairy roots with phosphate buffer, and the protein concentrations were then measured using the Pierce™ BCA protein assay kit (Thermo Fisher Scientific, Shanghai, China). The activities of CAT, SOD, and POD in the above liquid samples were then determined using the CAT assay kit, SOD assay kit, and POD assay kit, respectively, according to the product instructions.

### 2.8. Statistical Analysis

The experiments including the determination of root growth, Cd accumulation, and enzyme activities were performed in triplicate and the results were the means of triplicate samples ± S.D. The data obtained from the experiments were analyzed using IBM’s SPSS 20 software by one-way analysis of variance (ANOVA). Values followed by different letters are significantly different at *p* < 0.05 according to Duncan’s test [10].

## 3. Results

### 3.1. Development of Solanum nigrum L. Hairy Roots Expressing Heterologous IRT1

**Figure 1:**
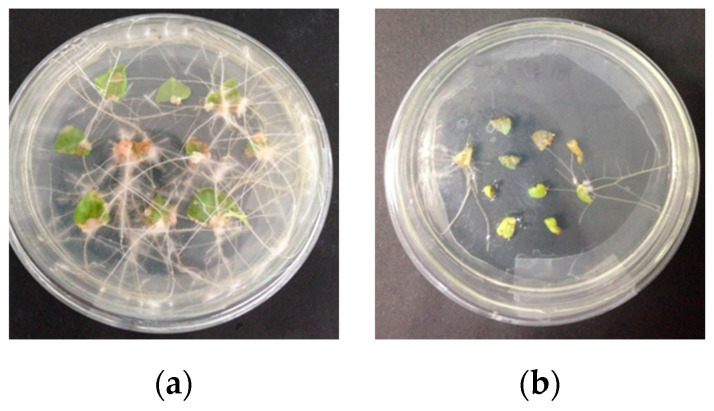
Photographs of the hairy roots of *Solanum nigrum* L. at 21 d induced with wild-type *Agrobacterium rhizogenes* ATCC15834 (**a**) and recombinant *Agrobacterium rhizogenes* ATCC15834 containing the *IRT1* gene (**b**).

Wild-type and transgenic hairy roots were induced via *Agrobacterium rhizogenes ATCC15834* in the MS medium and the results are shown in Figure 1. The induction of wild-type hairy roots was slightly faster than that observed in the case of transgenic hairy roots. 

**Figure 2:**
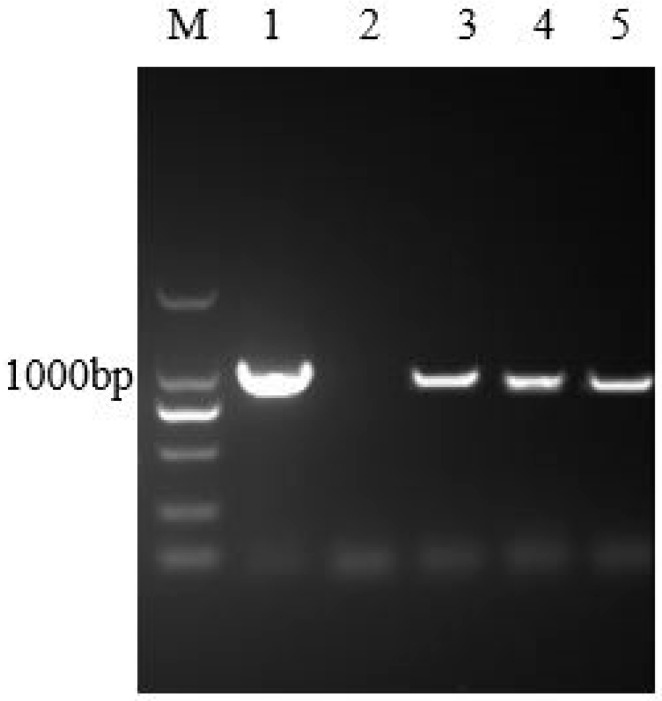
Identification of the transgenic hairy roots of *Solanum nigrum* L. using polymerase chain reaction (PCR). M: DNA marker 2000; Lane 1: the *pUC57* plasmid; Lane 2: sterile water; Lane 3~5: DNA extracted from transgenic hairy roots of *Solanum nigrum* L. expressing the *IRT1* gene.

The results of PCR showed that the target band appeared in the case of transgenic hairy roots (Figure 2). Furthermore, the results of the DNA sequencing indicated that the corresponding sequence was in complete accord with the *IRT1* gene sequence. The above results demonstrated that the heterologous *IRT1* gene was successfully transferred to the genome of *Solanum nigrum* L. hairy roots. Thereafter, the preliminary results of the western blot hybridization with Fe^2+^ transporters indicated the successful expression of the IRT1 protein in the transgenic hairy roots (Figure S2).

### 3.2. Growth of the Wild-Type and Transgenic Hairy Roots under Cd Stress

**Figure 3:**
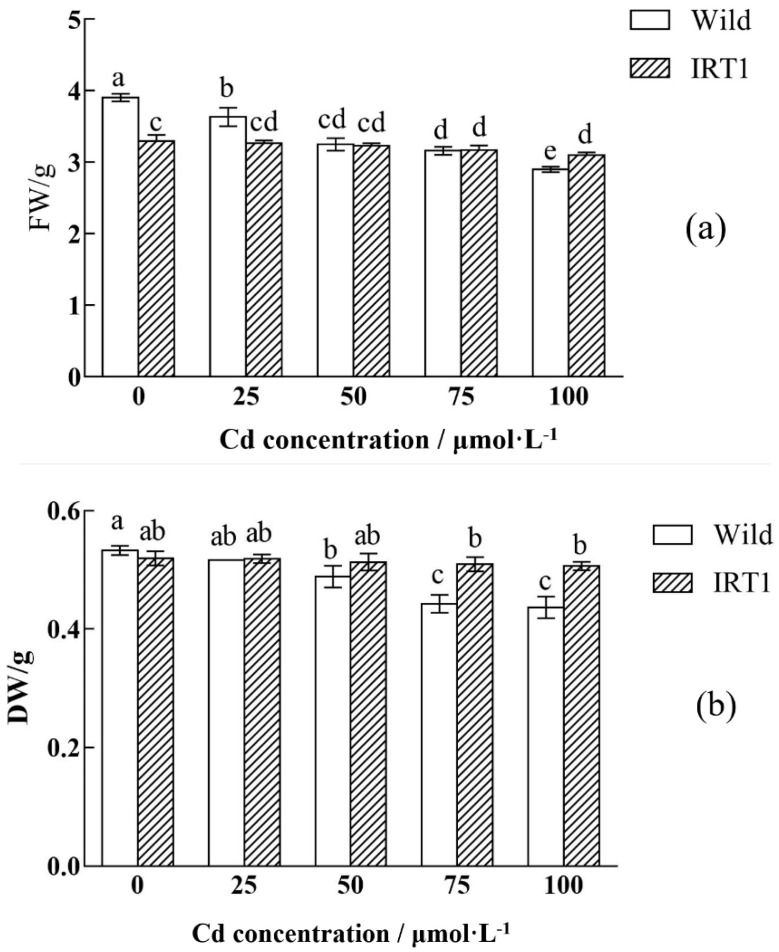
Biomass accumulation of the wild-type and transgenic hairy roots of *Solanum nigrum* L. growing in MS medium with or without Cd stress. (**a**) FW: fresh weight; (**b**) DW: dry weight. Values of biomass accumulation in this figure followed by different letters (i.e., a, b, c, and d) are significantly different at *p* < 0.05 according to Duncan’s test. The FW and DW were calculated based on biomass accumulation in 50 mL MS medium per flask.

To investigate the relationship between *IRT1* expression and Cd tolerance, the transgenic hairy roots constitutively expressing *IRT1* were subjected to Cd stress assay. After being incubated in the MS liquid medium without Cd for 35 days, the transgenic hairy roots of *Solanum nigrum* L. showed a comparable biomass accumulation compared to that observed in the case of wild-type hairy roots (0.520 g vs. 0.533 g) (Figure 3). 

**Figure 4:**
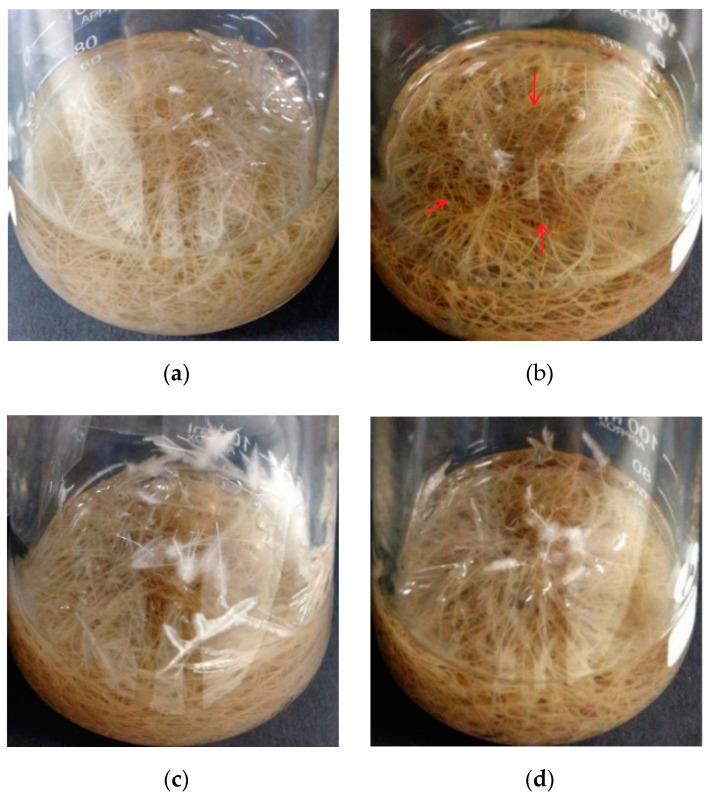
Photographs of the wild-type and transgenic hairy roots of *Solanum nigrum* L. growing in MS medium with or without Cd stress. (**a**): Wild-type hairy roots, 0 μM CdCl_2_; (**b**): wild-type hairy roots, 100 μM CdCl_2_; (**c**): transgenic hairy roots, 0 μM CdCl_2_; (**d**): transgenic hairy roots, 100 μM CdCl_2_.

The biomass of the wild-type hairy roots growing in the medium with 50 and 100 μM CdCl_2_ decreased by 9% and 22%, respectively (Figure 3), indicating significant growth inhibition under Cd stress (*p* < 0.05). Serious growth inhibition was not observed in the case of the transgenic hairy roots, and there was no big difference (Figure 3b, *p* < 0.05) between biomass accumulation (DW) during the cultivation without and with Cd stress, even in the presence of 100 μM CdCl_2._ The accumulated biomass of the transgenic hairy roots after 35 days of cultivation reached 0.507 g, against 0.437 g of that observed in the wild-type control. Wild-type hairy roots in the presence of high Cd stress (100 μM CdCl_2_) became slightly brown in color (the part with red arrow) (Figure 4b). In the transgenic hairy root cultures, the browning phenomenon under Cd stress was relatively weak (Figure 4d). 

### 3.3. Cd Accumulation in the Wild-Type and Transgenic Hairy Roots under Cd Stress

The hairy roots were collected for determining Cd content and the results are shown in Table 1.

As shown in Table 1, there was no significant difference in the Cd accumulation when two kinds of hairy roots were cultivated in the medium supplemented with 50 μM CdCl_2_. In the presence of higher Cd stress (100 μM CdCl_2_), enhanced Cd accumulation was carried out in the transgenic hairy roots compared to the control (886.8 μg/g vs. 745.0 μg/g).

### 3.4. ROS Release and Cell Damage in the Wild-Type and Transgenic Hairy Roots under Cd Stress

**Figure 5:**
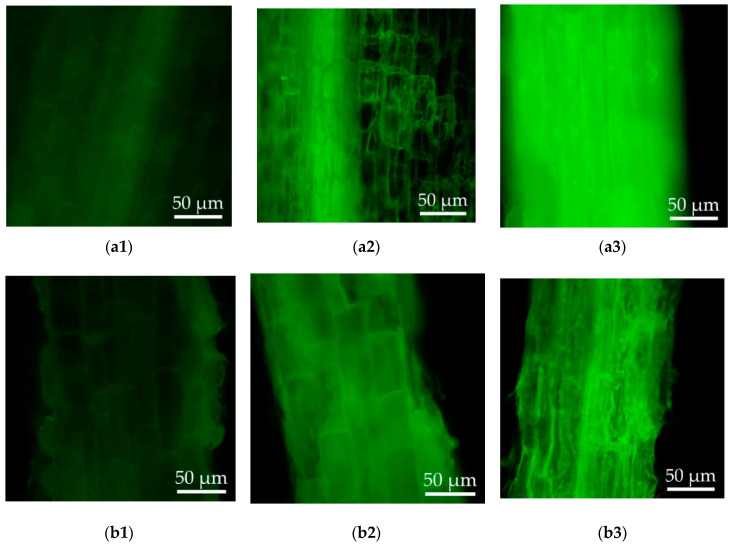
Reactive oxygen species (ROS) detection of the wild-type and transgenic hairy roots of *Solanum nigrum* L. growing in MS medium with or without Cd stress. (**a1**): Wild-type hairy roots, 0 μM CdCl_2_; (**a2**): wild-type hairy roots, 50 μM CdCl_2_; (**a3**): wild-type hairy roots, 100 μM CdCl_2_; (**b1**): transgenic hairy roots, 0 μM CdCl_2_; (**b2**): transgenic hairy roots, 50 μM CdCl_2_; (**b3**): transgenic hairy roots, 100 μM CdCl_2_.

Thereafter, to compare the cellular oxidative stress and cell damage under Cd stress between the wild-type and transgenic hairy roots, the levels of intracellular ROS and cell damage were studied. The DCFH could be oxidized by the intracellular ROS and converted into DCF with green fluorescence. The levels of ROS in the hairy roots can be reflected by observing the intensity of fluorescence. As shown in Figure 5, faint green fluorescence and cell wall structures were observed in both the wild-type and transgenic hairy roots of *Solanum nigrum* L. growing in the MS medium without CdCl_2_ (Figure 5a1,b1). Increased ROS levels were induced in the hairy roots under Cd stress. Compared to the wild-type hairy roots, the transgenic lines demonstrated reduced ROS level during middle Cd stress (50 μM CdCl_2_). In response to high Cd stress (100 μM CdCl_2_), cell wall boundaries became blurrier and much higher ROS was accumulated in the wild-type hairy roots than the transgenic lines (Figure 5a3,b3).

**Figure 6:**
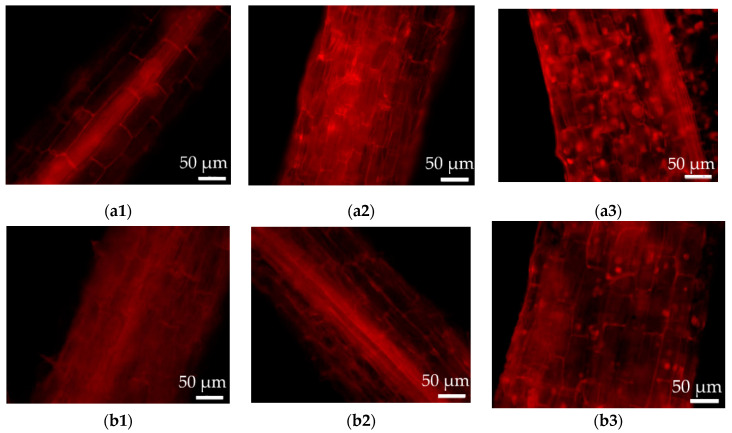
Cell damage observation by propidium iodide (PI) staining of the wild-type and transgenic hairy roots of *Solanum nigrum* L. growing in MS medium with or without Cd stress. (**a1**): Wild-type hairy roots, 0 μM CdCl_2_; (**a2**): wild-type hairy roots, 50 μM CdCl_2_; (**a3**): wild-type hairy roots, 100 μM CdCl_2_; (**b1**): transgenic hairy roots, 0 μM CdCl_2_; (**b2**): transgenic hairy roots, 50 μM CdCl_2_; (**b3**): transgenic hairy roots, 100 μM CdCl_2_.

The above results were further proved by the PI staining of the hairy roots (Figure 6), in which cell damage can be recorded by the red fluorescence intensity. As shown in Figure 6, the cell damage was present in both wild-type and transformed hairy roots. Much stronger cell damage was observed in the case of wild-type hairy roots growing in the medium with 100 μM CdCl_2_ (Figure 6a3), as compared to that without Cd addition (Figure 6a1). The results of the PI staining also revealed less fluorescent spots (i.e., less cell damage) in the transgenic hairy roots (Figure 6b3) under high Cd stress (100 μM CdCl_2_) relative to the wild-type lines (Figure 6a3). 

### 3.5. Antioxidant Enzyme Activities in the the Wild-Type and Transgenic Hairy Roots under Cd Stress 

**Figure 7:**
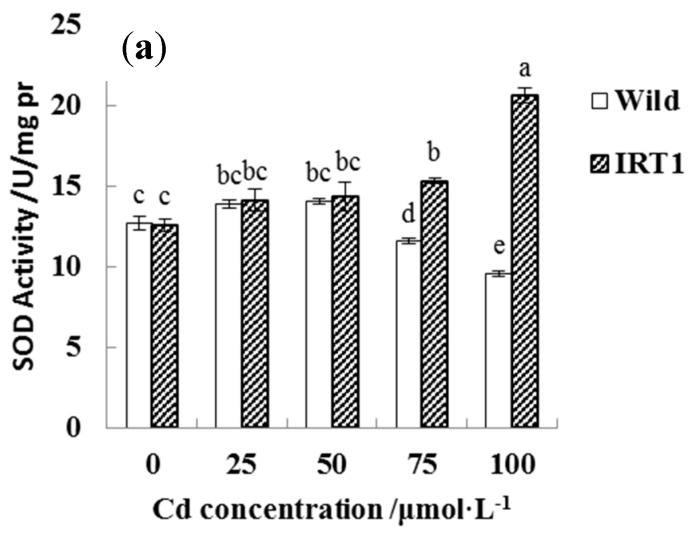

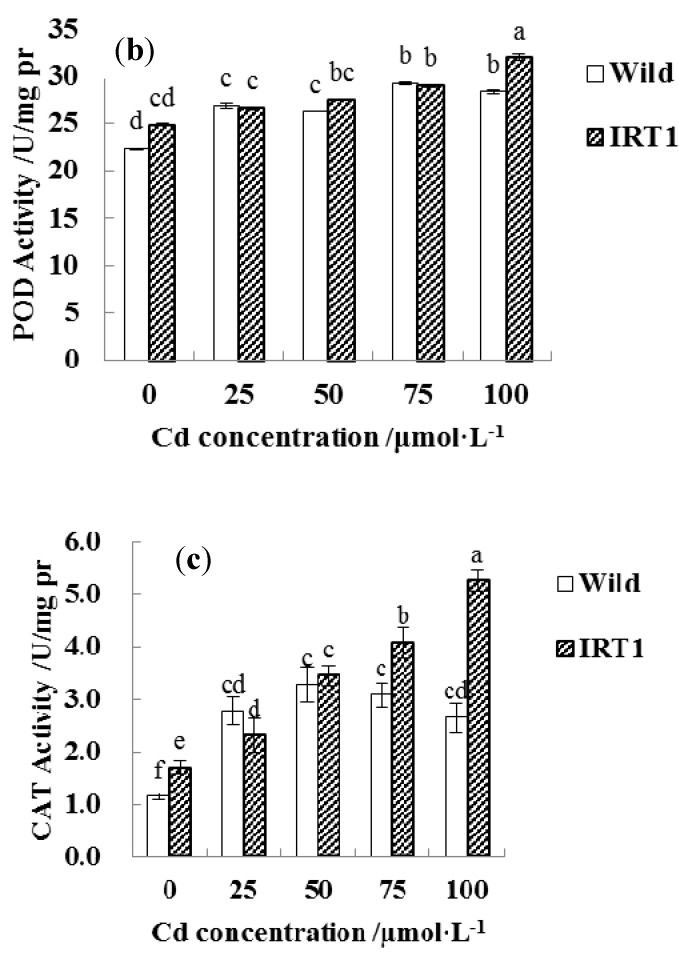
Antioxidant enzyme activities in the wild-type and transgenic hairy roots of *Solanum nigrum* L. growing in MS medium with or without Cd stress. (**a**): superoxide dismutase (SOD); (**b**): peroxidase (POD); (**c**): catalase (CAT). Values of enzyme activities in this figure followed by different letters (i.e., a, b, c, d, e, and f) are significantly different at *p* < 0.05 according to Duncan’s test.

For evaluating Cd stress response in the root tissues, different assay kits were employed to determine the activities of antioxidant enzymes including CAT, POD, and SOD (Figure 7). In the case of the wild-type hairy roots, as the CdCl_2_ concentrations in the medium increased from 0 to 50 μM, SOD, CAT, and POD activities showed a slight increase. In the presence of higher Cd stress (75~100 μM CdCl_2_), SOD and CAT activities even had a significant decrease, while POD remained at a stable level. 

Interestingly, the transgenic hairy roots demonstrated significantly increased SOD, CAT, and POD activities under Cd stress, even in the presence of 100 μM CdCl_2_. As shown in Figure 7a–c, SOD and CAT activities in the transgenic hairy roots under the highest Cd stress (100 μM CdCl_2_) increased by nearly 100% than the wild-type lines.

## 4. Discussion

It is important to study the Cd tolerance and the corresponding molecular mechanism of different Cd-hyperaccumulating plants. In the present study, *Solanum nigrum* L., a hyperaccumulator of Cd, was selected as a model plant system to study the potential application of the *IRT1* gene for efficient phytoremediation of Cd pollution. The transgenic hairy roots of *Solanum nigrum* L. expressing the *IRT1* gene were successfully obtained via the *Agrobacterium tumegaciens*-mediated method.

Interesting, the transgenic hairy roots exhibited superior growth patterns and improved Cd accumulation under Cd stress compared to the wild-type hairy roots. Weak red fluorescence was observed in the PI staining results of wild-type and transgenic roots of *Solanum nigrum* L. in the absence of Cd stress, which could be due to slight cell damage and/or normal cell death in this normal growing condition [30,31]. In the present of high Cd stress (100 μM CdCl_2_), much stronger ROS release and cell damage were recorded in the wild-type hairy roots. The transgenic lines expressing the *IRT1* gene exhibited increased antioxidant enzyme activities (SOD, POD, CAT), lower ROS levels, and less cell damage compared to the wild-type control. Previous studies suggest that Cd is a phytotoxic metal pollutant that induces ROS release and inhibits the growth of plants [2,8,15]. High Cd stress and prolonged exposure produce cell damage (e.g., lipid peroxidation, protein oxidation, and enzyme inhibition) and finally induces cell death due to oxidative stress [2,8,15]. Plants may improve their tolerance by complex responses involving anti-oxidative mechanisms, Cd compartmentalization inside the cell wall and/or vacuole, and so on [2,10,15]. On one hand, the present results clearly show that the functional activity of the *IRT1* transgene is capable of improving anti-oxidative protection against external Cd stress and helps the transgenic hairy roots to mitigate the Cd toxicity. Lower antioxidant enzyme activities, higher ROS levels, and serious cell apoptosis in the roots of wild-type lines under Cd stress, as shown in Figure 5, Figure 6 and Figure 7, further proved the role played by the *IRT1* gene on the anti-oxidative response in the transgenic hairy roots of *Solanum nigrum* L. Previous studies reported that the synthesis of some antioxidants (e.g., glutathione, GSH) in the hairy roots exposed to external Cd stress may be increased by up-regulating expression of the related genes [10]. The expression of genes related to key enzymes of GSH metabolism, including ROS scavenging (e.g., glutathione peroxidase, GPX and glutathione *S*-transferase, GST) and GSH regeneration from oxidized glutathione (e.g., glutathione reductase, GSR), increased in response to the stress of various heavy metals (e.g., Ni, Cd etc.) [10,32,33,34].

On the other hand, the transgenic *IRT1* hairy roots of *Solanum nigrum* L. may modulate Cd transportation and distribution by expanding expression levels and territories for IRT1 in roots, hence improving their Cd accumulation and tolerance. The modulation of Cd distribution in plants (e.g., sequestrating metal in organelles or vacuoles, long-distance transport from root to shoot, and then leaf vacuoles) plays an important role in enhanced Cd accumulation [35]. Heavy metal transporter *IRT1* showed higher expression in either Cd-treated or iron-deficiency *Solanum nigrum* L. roots (a high Cd accumulator) than in *Solanum torvum* roots (a low Cd accumulator), which may be responsible for differential uptake and redistribution in the two *Solanum* species [35]. In this study, the transgenic hairy roots were obtained by expressing *IRT1* from *Arabidopsis thaliana*. Therefore, transformed plants have their own *IRT1* plus *IRT1* gene from *Arabidopsis thaliana*. Extra expression of *IRT1* in the hairy roots of *Solanum nigrum* L., together with its own great uptake capacity of Cd, might further improve its ability to promote Cd translocation. Previous studies also showed that plants (*Arabidopsis thaliana*) overexpressed *IRT1* accumulated higher levels of Cd and Zn than wild-type plants, indicating that *IRT1* is responsible for the uptake and transport of Cd [14]. Over-expression of the *MxIRT1* gene increased Fe, Zn, and Cd content in in rice seeds by about 1.4~3 times [36]. Moreover, IRT1 protein expressed from the transgene accumulated in both roots and leaves, while endogenous IRT1 protein is barely detected in the leaves of wild-type plants [37]. Expression of *Arabidopsis thaliana IRT1* in plants leads to constitutive accumulation of IRT1 protein, which is found in *trans*-Golgi network/early endosomes of root hairy cells regardless of iron supply to roots and cycles to the plasm membrane to significantly enhance iron and metal uptake (e.g., more than 10-fold higher Zn, Co, and Mg levels than the wild-type lines) [37]. Therefore, enhanced Cd accumulation (Table 1) in the present transgenic hairy roots of *Solanum nigrum* L. expressing *IRT1* likely results from expanded expression territories for IRT1 protein, besides its expression levels, compared to wild-type line.

To summarize, all above factors including antioxidants (e.g., GSH and AsA), metal transporters, and metal chelator may produce an integrated protection for plants from the Cd stress [10,15,38,39]. Cd accumulation is a complicated process, which is regulated by the integrated gene modulation network. Together with previous works, the results in the present study provide important information for the development of transgenic plants with hyperaccumulating ability by overexpressing one of these candidate genes or their combination.

When considering the diversity of metal-contaminated soils, more studies are needed to understand the real mechanism of the metal transport, competitive interactions, and tolerance to multi-metal toxicities in these plants under heavy metal stress to help improve their accumulating abilities further in order to target heavy metals. In addition, the use of these hyperaccumulating plants in combination with other physical, chemical, and/or biological ways, including application of plant growth regulators (e.g., citric acid), inorganic amendments (e.g., silicon, and elemental sulfur), organic amendments (e.g., manure), and microbes [8], could also enhance the remediation and shorten the time required.

## 5. Conclusions

Growth inhibition, oxidative damage, and cell death are widely induced by heavy metal stress. The results in the present work clearly show that the expression of *IRT1* in the hairy roots of *Solanum nigrum* L. provides important protection against Cd-induced oxidative stress. Transgenic hairy roots show significantly superior root growth, increased antioxidant enzyme activities, decreased ROS levels, and less cell apoptosis compared to the wild-type line. As a result, the expression of *IRT1* in the hairy roots improved the Cd accumulation level by 19% compared to the wild-type line. The total amounts of Cd accumulation in the transgenic line will be higher when considering that biomass accumulation obtained another 16% increase than the control. These results provide useful information for improving our understanding of the Cd tolerance of *Solanum nigrum* L. and prove that the *IRT1* gene is a valuable candidate for engineering heavy metal stress tolerance in plants. It is expected that expressing additional genes (e.g., metal chelator genes and *gshB* gene) in the transgenic *IRT1* hairy roots may produce a better hyperaccumulator that can extract and accumulate heavy metal from the contaminated water and soil even more effectively. 

## Figures and Tables

**Table 1:** Accumulated Cd levels in the wild-type and transgenic hairy roots of *Solanum nigrum* L.

CdCl_2_ Concentrations (μM)	Cd Content in Transgenic Hairy Roots (μg/g)	Cd Content in Wild-Type Hairy Roots (μg/g)
0	0	0
50	365.4 ± 2.6 ^c^	367.7 ± 21.3 ^c^
100	886.8 ± 1.0 ^a^	745.0 ± 10.5 ^b^

Note: The experiments were performed in triplicate (n = 3). Values followed by different letters are significantly different at *p* < 0.05 according to Duncan’s test.

## Supplementary Materials

The following are available online at https://www.mdpi.com/2075-1729/10/12/324/s1, Table S1. Primers’ sequences of PCR, Table S2. PCR cycling conditions, Figure S1. Schematic representation of the T-DNA region of the recombinant plasmid pRI101-IRT1 based on plasmid pRI101-AN, Figure S2. Western blot test of transgenic hairy roots of *Solanum nigrum* L. Lane 1~3: the IRT1 protein observed in the transgenic hairy roots of *Solanum nigrum* L.
