# Supplementary material for: Enhanced Cadmium Accumulation and Tolerance in Transgenic Hairy Roots of Solanum nigrum L. Expressing Iron-Regulated Transporter Gene IRT1
Source: Life (Basel). 2020 Dec 3;10(12):324. doi: 10.3390/life10120324 (PMC7761695; doi:10.3390/life10120324)
Supplement: Supplementary file 1 [file life-10-00324-s001.pdf]

life

Supplementary Material of Enhanced Cadmium Accumulation and Tolerance in Transgenic Hairy Roots of *Solanum Nigrum* L. Expressing Iron-Regulated Transporter Gene *IRT1*

Table S1. Primers' sequences of PCR

Primers	Sequences
Forward primer	ATG GCT TCA AAT TCA GCA CTT CTC
Reverse primer	TTA AGC CCA TTT GGC GAT AAT CG

Table S2. PCR cycling conditions

Operation steps	Conditions
Denaturation step	94 °C, 10 min
Cycles	35 cycles: 94 °C, 1 min; annealing, 56 °C, 1 min, and elongating, 72 °C, 1 min
Elongation	72 °C, 10 min

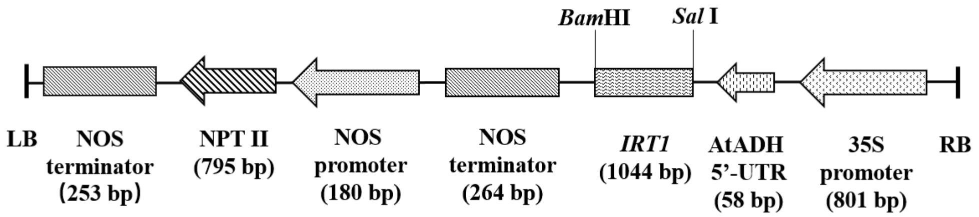

Figure S1. Schematic representation of the T-DNA region of the recombinant plasmid pRI101-*IRT1* based on plasmid pRI101-AN.

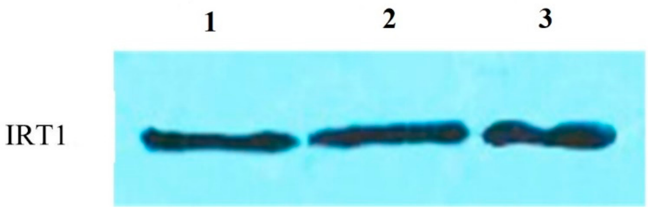

Figure S2. Western blot test of transgenic hairy roots of *Solanum nigrum*. L. Lane 1~3: the IRT1 protein observed in the transgenic hairy roots of *Solanum nigrum* L.

Publisher's Note: MDPI stays neutral with regard to jurisdictional claims in published maps and institutional affiliations.

© 2020 by the authors. Submitted for possible open access publication under the terms and conditions of the Creative Commons Attribution (CC BY) license (http://creativecommons.org/licenses/by/4.0/).
